# Abroad and at Home

**Published:** 1890-08-16

**Authors:** 


### The Hospital

A Weekly Journal of

Science, Medicine, Nursing, and Philanthropy.

*For Hospitals, Asylums, Medical Practitioners, Students, Nurses, Families, Parishes, Congregations, and the Charitable Public.*

Vol. VIII.—No. 203.] [August 16, 1890.

### Abroad and At Home.

Englishmen, or rather Britons, are proud of their fellow countrymen who have risen to fame, even when they belong to professions so little understood by the public as those of medicine and surgery. Sir James Paget and Sir Joseph Lister were among the heroes of the hour at the International Congress at Berlin last week. The fame of those two, as of several other British physicians and surgeons, has extended to the remotest bounds of the civilized world. It was in every way right and proper that British medicine and surgery should be worthily represented at the Universal Congress of Healers. The men of this country possess endowments of character peculiar to themselves, and which serve important purposes on occasions when representatives of all nations stand face to face and take moral and intellectual measure of each other. The educated Briton is a stern kind of man and not too flexible. He has the habit of looking very directly at a thing. He may sometimes fail to see all that is to be seen; but he seldom adds anything to the actual facts by the undue liveliness of his imagination. What he sees he knows and talks confidently about; what he does not see he generally holds to be doubtful, and asks for evidence. He stands by the actual and is not easily moved, either by an impatient eagerness for notoriety, or by a meek, if amiable, desire to agree with his cultured fellow-men.

Mental stuff of the kind that the best British doctors are made of has great value everywhere. The German theorists, the precisionists of France, and the daring innovators of America are all conscious that their work, when submitted to the stern and sober scientific Puritans of this country, may be found of much less real and enduring value than they had thought it. To our countryman is paid the respect which is always yielded to those who plant their feet firmly on the rock of evident and demonstrated fact. In no department of human activity is this peculiar temper of mind more essential than in physiology, morbid activity, and therapeutics. In all those fields of science baseless theorising and hasty generalisation are alike fatal to progress and injurious to the world. British medicine does not play the part of a barrier to progress, but of a moderator of the pace. It aims at the definite goal of skilled and successful healing; and when compared either with French, German, or American medicine, it is found to be, on the whole, well in advance. We do not speak now of exceptional scientific discoveries like those of Koch, of new operational departures like that of McDowell, or of brilliant experimental inductions like those of Charcot, all of which are worthy of the highest honour which universal science can bestow. But we refer to that general competency in the management of disease, and that high average of success in its treatment which so honourably distinguish the rank and file of the medical profession throughout the British Empire. It is the great honour of our country to have scientific leaders who rank second to none in the world, and an army of like-minded followers, most of whom have the leader's capacity if opportunity were favourable or necessity should arise.

The International Medical Congress at Berlin followed in quick succession upon the annual meeting of the British Medical Association at Birmingham. At that meeting, also, some of the ablest and most eminent of British physicians and surgeons were present, and with admirable skill and earnestness strove to infuse into the minds of their less generally known brethren the highest ideals of practice and the noblest enthusiasms of progress. Those who met together at Birmingham and shared in the work of the week could not but feel that the occasion was one of mutual inspiration, of enlightenment by contact of mind with mind, and of the deepening of those feelings of brotherly sympathy and regard which add strength to a great profession, and give elevation to its work. But certain well-known persons were conspicuous by their absence. It is not, perhaps, necessary or desirable to inquire why they shunned the meeting of their medical fellows at Birmingham, and sought that at Berlin. What, however, it is desirable to bear in mind is that the British Medical Association is the only body which can really lay claim to being representative of the medical profession in this country. That being so, the secession from its ranks of a number of influential persons is a fact of great importance both to it and to them. Such a secession may conceivably be justified. There are not wanting historical parallels which have commanded the sympathy and adhesion of vast numbers of persons of the highest intellect and character.

But does any body pretend that the British Medical Association as a body has been guilty of such flagrant departures from honourable medical tradition as to render it no longer worthy of being the common meeting ground of men of every variety of opinion and status in the profession? The simplest statement of the truth is that the British Medical Association still claims among its members a preponderant majority of physicians and surgeons of the highest rank and the worthiest character in the British Empire. Compared with the many who are left, the few who have gone are a mere handful; and even the handful themselves will not venture to say that the present leaders of the loyal many are inferior to them either in professional eminence or in personal worth. It is manifest that there has been a schism within the ranks of medicine; and though, as we have said, it is possible to imagine a justifiable schism, yet the very fact that so many men of the first rank still remain loyal to the Association, seems to preclude the possibility of adequate justification in the present instance.

The bulk of the profession have just ground of complaint against those who have cut themselves off from the great confraternity of medical brethren. It is to be remembered that the profession is a brotherhood holding a peculiarly isolated and responsible position. It is to be remembered also that physicians and surgeons are but men, liable to all the errors of judgment and faults of character which appertain to other men. Is it a generous or worthy act for a few of the leaders of an army to desert those whose suffrages and approval have raised them to eminence, on a mere difference of opinion? It can hardly be doubted that a serious mistake has been made; and it is equally evident that a right spirit would prompt to a retracing of the unwise steps. Achilles sulked obstinately in his tent when he felt himself injured and aggrieved. But he did not sulk there for ever. He was still a Greek, and the Trojans were his foes; and when he had buckled on his armour again he gave convincing proof of the unspoiled heroism of his character. British medicine needs all the inspiration it can get from the unstinted sympathy and loyalty of its leaders. A pharisaic assertion of any kind of superiority is fatal to sympathy. Whatever a few persons may think, there is no doubt at all that, tried by any test which an experienced man of the world or a sober moralist would approve, the British Medical Association is as worthy of the confidence and loyal regard of the whole medical profession to-day as it has ever been from the commencement of its history, or as the Berlin Congress with which we are comparing it. If that be so—and it certainly is—what must be the verdict of good sense and right feeling upon those who, on the sole ground of a difference of opinion upon a mere personal question, deserted the Association, and refused to reconsider their position? The soberest, the wisest, and the worthiest may err; but an error becomes a fault when it is perceived and persisted in.

